# Dual access to the fluid networks of colloid-stabilized bicontinuous emulsions through uninterrupted connections†

**DOI:** 10.1039/d4mh00495g

**Published:** 2024-07-25

**Authors:** Mariska de Ruiter, Meyer T. Alting, Henrik Siegel, Martin F. Haase

**Affiliations:** a Van’t Hoff Laboratory of Physical and Colloid Chemistry, Department of Chemistry, Debye Institute for Nanomaterials Science, Utrecht University Utrecht The Netherlands m.f.haase@uu.nl

## Abstract

Large surface areas are important for enhancing mass and energy transfer in biological and technological processes. Bicontinuous interfacially jammed emulsion gels (bijels) increase the surface area between two fluids by intertwining them into particle stabilized networks. To facilitate efficient mass and energy exchange *via* the bijels’ high surface area, the fluid networks need to be connected to their respective bulk phases. Here, we generate bijels between two bulk fluids and investigate the connections the bijel makes. We analyze these connections by investigating the colloidal stability, interfacial rheology and mass transfer dynamics during bijel formation. To this end, we employ confocal and electron microscopy, as well as dynamic light scattering, pendant drop analysis, electrophoretic mobility measurements and diffusion simulations. We find that the connections the bijel makes to the bulk fluid can be disrupted by severe colloidal aggregation and interruptions of the bicontinuous fluid network. However, the addition of alcohol to the bulk fluid moderates aggregation and allows undisturbed fluid network formation, facilitating open connections between bijel and bulk fluid. The unprecedented control of bijel pore connections from this research will be crucial for the application of bijels as separation membranes, electrochemical energy storage materials and chemical reactors.

## Abstract 2

Bijels intertwine two fluids into stable, bicontinuous networks. This work introduces dual pore connections for bijels to two bulk phases, a unique architecture with potentials to overcome diffusion limitations for batteries and separation membranes.

## Graphical abstract

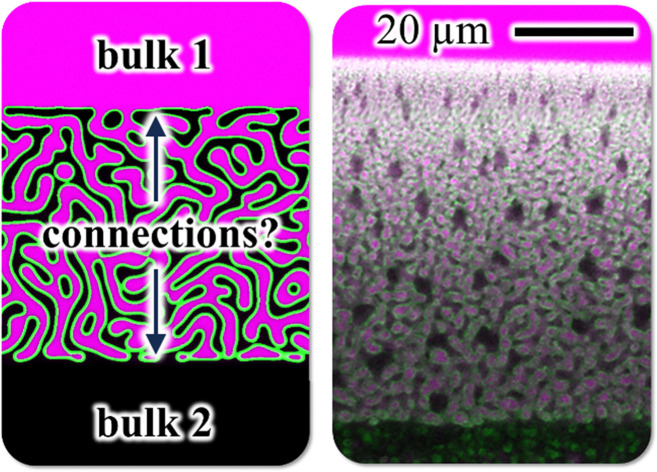

**New concepts**

Bijels are particle-stabilized networks of two fluids with potential applications in batteries, separation membranes, and chemical reactors. Although prior research has advanced the understanding of bijel synthesis and applications, only one of the two fluid networks has been connected to its respective bulk phase. This manuscript introduces bijels with open and uninterrupted connections to two different bulk phases, a crucial architecture for realizing many of their application potentials. Bijels with dual connections not only increase the surface area between two bulk phases but also provide separate access to each microscopic channel network of the bijel. This study finds that the boundary between bijels and their surroundings can be obstructed by severe colloidal aggregation and interruptions in the bicontinuous fluid network. Open and uninterrupted connections are essential for molecular exchange between the bijel and its environment. To achieve open connections, we control phase separation kinetics and colloidal self-assembly using well-defined chemical gradients during bijel fabrication. Our findings, supported by complementary experiments and simulations, offer significant insights for the broader material science community. The open pore connections developed in this work are crucial for using bijels as high-surface area materials that overcome mass-transfer limitations in batteries, separation membranes, and chemical reactors.

## 1. Introduction

The concept of surface area holds profound significance in both biological and technological realms, playing a pivotal role in facilitating efficient processes essential for life and engineering. A notable example in biology is evident within the intricate structure of the alveoli, the microscopic air sacs nestled within the lungs.^1^ Here, nature has ingeniously crafted a vast surface area optimized for the crucial exchange of gases, where oxygen seamlessly diffuses into the bloodstream while carbon dioxide exits with equal ease. This design ensures the vital supply of oxygen necessary for cellular respiration, underscoring the indispensable role of high surface areas in biological functions. Similarly, in the realm of technology, the strategic augmentation of surface area finds application in the form of cooling ribs meticulously integrated into electronic devices.^2^ By expanding the surface available for dissipation of heat, these cooling ribs enhance thermal management, thereby safeguarding the efficiency and longevity of electronic components. Thus, whether in the intricate mechanisms of life or the sophisticated circuits of technology, the maximization of surface area emerges as a fundamental principle indispensable for enhancing diffusive exchange of matter and heat.

Engineers employ machining, extrusion or additive manufacturing to increase the surface area of electronic devices, heat exchangers, and catalytic converters. Even higher surface areas, that are needed for microfluidics and sensors, are obtained by microfabrication *via* lithography, micro-molding, or micro-electrochemical machining.^3^ However, if specialized equipment is unavailable, materials with high surface areas can also be fabricated *via* self-assembly processes.^4,5^ Bicontinuous interfacially jammed emulsion gels (bijels) are self-assembled soft materials with enlarged surface area that find applications in catalysis,^6,7^ electrochemical energy storage,^8–11^ healthcare materials,^12–14^ radiative cooling^15^ and membrane separations.^16,17^ Bijels intertwine two immiscible liquids in an arrested bicontinuous network by stabilizing their interface with colloidal particles.^18,19^ The fluidic networks of bijels facilitate intimate contact between two liquids without interrupting their connectivity. This bicontinuous arrangement in bijels holds promise for applications that benefit from enlarged surface areas between two bulk phases, for example to increase the interaction between reagents during chemical reactions.^20–22^

The recent review article by di Vitantonio *et al.* concludes “connecting each domain [of a bijel] to an external source(s) or bath(s) to enable delivery and retrieval of reagents needs to be addressed to truly take advantage of the bicontinuous morphology…”.^23^ But, how can both channel networks of bijels be connected to their respective bulk phases? In their classical review article, Cates and Clegg suggest: “Intriguingly, a bijel device would ‘plumb in’ automatically to adjacent bulk phases of the two fluids: a bulk phase of either fluid should connect, without interface or other obstruction, to the matching bicontinuous domain.”^24^ Indeed, bijels have been connected to a single bulk phase before, as demonstrated by the diffusion of monomers^25^ and dyes.^26^ However, to fully utilize the large surface area of the bijel, the interface needs to be accessible from both bulk phases. The complete potential of the bijel for applications such as dialysers,^27^ batteries,^28^ electrolysers^29^ and fuel cells^30^ can be realized only when fluids, electricity, or heat can enter both channel networks of the bijel.

Here, we test the hypothesis that the water and oil channels of a bijel can be simultaneously connected to a bulk water and a bulk oil phase *via* solvent transfer induced phase separation (STrIPS).^26,31–35^ To this end, we employ fluidic devices to fabricate hollow bijel fibers. *Via* these devices, we contact the bijel precursor mixture with two separate continuous phases simultaneously. We vary the composition of the continuous water phase and investigate the effect on the bijel architecture using scanning electron microscopy and confocal laser scanning microscopy. Measurements of the colloidal stability provide important insights about the channel openings to their respective bulk phase. Analysis of liquid miscibility, interfacial rigidity and diffusion simulations explain the bijel's network structure next to the bulk water phase. We find that connecting the bijel's water channel network to the bulk water phase requires control over colloidal stability and phase separation dynamics. To bulk oil phases, the bijel connections facilitate diffusive exchange of hydrophobic molecules, enabling postprocessing of the bijel.

## 2. Results and discussion

### 2.1 Fabrication of bijels with open pore connections

**Fig. 1:**
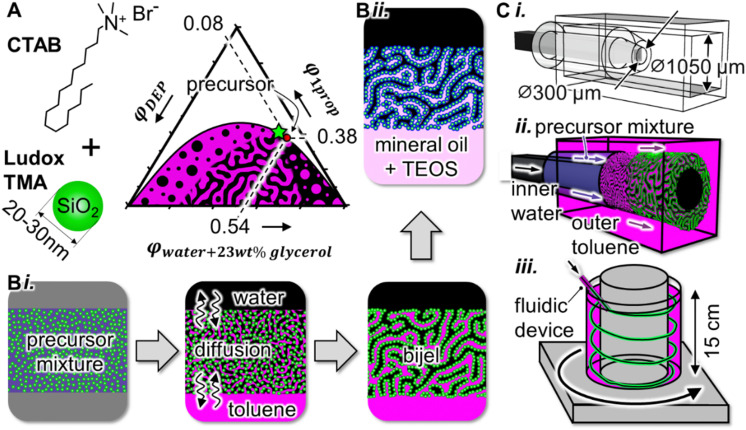
Hollow bijel fiber fabrication. (A) Chemical formula of CTAB, schematic depiction of a Ludox TMA SiO_2_ nanoparticle, and phase diagram with binodal curve and critical point (green star). The schematic drawing below the binodal curve depicts regions of nucleation and spinodal decomposition. (B)-(i) Schematic illustration of bijel formation *via* STrIPS with both toluene and water bulk phases. (B)-(ii) Schematic of nanoparticle cross-linking with TEOS. (C)-(i) Computer drawings of devices composed of three coaxially aligned glass capillaries, (C)-(ii) illustration of hollow fiber synthesis, (C)-(iii) schematic of hollow fiber collection in toluene filled, rotating, cylindrical annulus.

Bijels are synthesized with a multicomponent, homogeneous precursor mixture. This mixture contains diethylphthalate (DEP), water, glycerol, 1-propanol, cetyltrimethyl ammonium bromide (CTAB) and Ludox TMA SiO_2_ nanoparticles. In the phase diagram of Fig. 1A, the composition of this liquid mixture (red dot) is located near the critical point (green star, see also Fig. S1, ESI†). Upon shifting the composition below the binodal curve, the mixture phase separates into water- and DEP(oil)-rich phases. During phase separation, CTAB functionalized nanoparticles self-assemble as a dense layer on the oil/water interface.^26,34^ This NP layer can arrest the phase separation *via* interfacial jamming, resulting in an interwoven network of oil and water channels.

In this research, we induce phase separation by diffusive mass-transfer between the bijel precursor mixture, a bulk toluene and a bulk water phase. As suggested by Cates and Clegg,^24^ we hypothesize that open bijel pore connections form when the phase separating liquid is miscible with the surrounding bulk phase. According to this hypothesis, open connections are created between the DEP-rich phase and bulk toluene, as well as between the water-rich phase and bulk water, as schematically shown in Fig. 1B(i).

Here, we expose the bijel precursor mixture simultaneously to water and toluene bulk phases *via* the fluidic device depicted in Fig. 1C. We assemble the fluidic device by coaxially aligning glass capillaries of square and round cross-sections (Fig. 1C(i) and Fig. S2, ESI†). The ends of these capillaries are tapered for flow focusing. An inner stream of water flows into a stream of the precursor mixture, which itself flows into an outer stream of toluene. These combined flows result in a cylindrical bijel shell around an aqueous core (Fig. 1C(ii)). After travelling for ∼1 cm in the fluidic device, the bijel shell enters a rotating reservoir of toluene and sinks to the bottom (Fig. 1C(iii)).

**Fig. 2:**
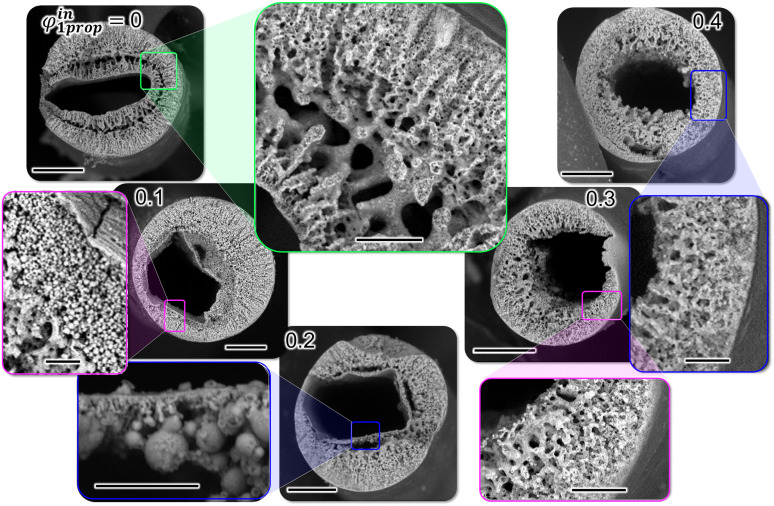
Hollow fiber structure analysis. SEM images of bijel fibers with inner water channels containing variable 1-propanol volume fractions *φ*^in^_1prop_. Scale bars of SEM images showing the full fiber cross-section: 100 μm and of images showing magnified views: 20 μm.

In the following, we investigate the connections the bijel makes to the inner water stream. To this end, we control the diffusive mass-transfer by varying the volume fraction of 1-propanol in the inner aqueous stream (*φ*^in^_1prop_). Scanning electron microscopy (SEM) is employed to probe the channel connections the bijel makes to the inner aqueous bulk phase. SEM characterization requires complete drying of the bijel shell, which results in the collapse of the bijel structure (Fig. S3, ESI†). Nevertheless, a stable imprint of the bijel silica scaffold can be attained upon cross-linking the particles. To this end, we exchange the toluene in the reservoir with a solution of 3 wt% tetraethyl orthosilicate (TEOS) in mineral oil. TEOS cross-links the particles in the bijel by SiO_2_ deposition *via* hydrolysis and condensation (Fig. 1B(ii)). Fig. 2 shows SEM images of TEOS cross-linked bijel structures of variable *φ*^in^_1prop_.

A rich variety of effects is observed when increasing *φ*^in^_1prop_. At *φ*^in^_1prop_ = 0, bicontinuous pore networks extend into the bijel shell from both inner and outer boundaries. Interestingly, the hollow fibers produced with *φ*^in^_1prop_ = 0.1 and 0.2 have inner water channels that are angular in shape. The angular water channel is surrounded by a thick, solid crust. High magnification scanning electron microscopy reveals that this crust is composed of densely packed nanoparticles (Fig. S4, ESI†). Below the crust, we find spherical microparticles that grow in size when *φ*^in^_1prop_ is increased from 0.1 to 0.2. Remarkably, for *φ*^in^_1prop_ = 0.3 and 0.4, no crust can be observed and the inner water channel is openly connected to the water network of the bijel. In the following, we discuss the various structures in Fig. 2 by analyzing the effect of *φ*^in^_1prop_ on the miscibility, the colloidal stability and the diffusion dynamics.

### 2.2 Structure formation mechanisms

**Fig. 3:**
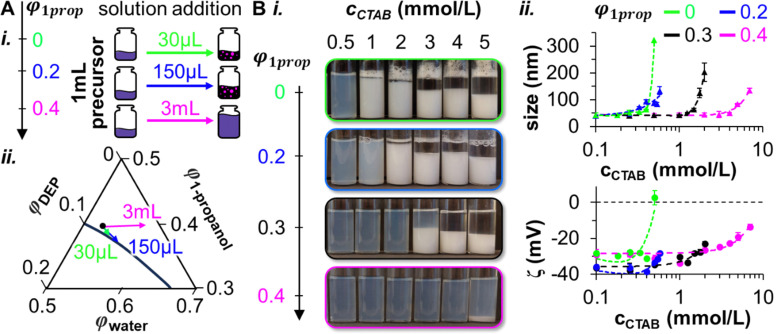
Miscibility and colloidal stability analysis. (A)-(i) Miscibility test between the bijel precursor mixture and aqueous phases of variable 1-propanol volume fractions *φ*_1prop_. (A)-(ii) Magnified phase diagram depicting compositional change upon adding different volumes of 1-propanol solutions of variable *φ*_1prop_ to the precursor mixture. (B)-(i) Photographs of aqueous dispersions of Ludox TMA nanoparticles at pH 3 with variable *φ*_1prop_ and CTAB concentrations *c*_CTAB_. (B)-(ii) Size measurements obtained *via* dynamic light scattering and zeta-potential (*ζ*) *via* electrophoretic mobility analysis of Ludox TMA dispersions (pH 3) at variable *φ*_1prop_ and *c*_CTAB_.

First, we discuss the miscibility of the bijel precursor mixture with the inner water channel in dependence of *φ*^in^_1prop_. To characterize the miscibility, we determine the volumes of aqueous solutions of variable *φ*_1prop_ required to induce phase separation in 1 mL of the bijel precursor mixture (see also Fig. S5, ESI†). For simplicity, in this experiment the aqueous solutions do not contain glycerol and the bijel precursor mixture does neither include nanoparticles nor CTAB. Fig. 3A(i) shows schematically that with increasing *φ*_1prop_, larger volumes of the aqueous solution need to be added to induce phase separation.

The experiment in Fig. 3A(i) shows that *φ*_1prop_ determines the miscibility of the precursor mixture with water. For *φ*_1prop_ = 0, 30 μL and for *φ*_1prop_ = 0.2, 150 μL need to be added to induce phase separation. Interestingly, for *φ*_1prop_ = 0.4, phase separation cannot be observed, irrespective of the added volume. These observations become plausible when analyzing the compositional change in the phase diagram of Fig. 3A(ii). With increasing *φ*_1prop_, more added solution volume is needed to move the composition below the binodal curve. However, for *φ*_1prop_ = 0.4, the composition never touches the binodal curve. Thus, an increasing *φ*^in^_1prop_ signifies better miscibility of the precursor mixture shell with the inner aqueous stream.

Next, we investigate the colloidal stability to gain further insights about the mechanisms controlling the shape and structure of the inner water channel. To this end, we analyze the aggregation of the nanoparticles in dependence of *φ*_1prop_. Fig. 3B(i) shows photographs of NP dispersions in water of variable *φ*_1prop_ and CTAB concentrations *c*_CTAB_. At *c*_CTAB_ = 0.5 mM, the NP dispersion appears in a blueish color, indicative of Tyndall scattering. However, as *c*_CTAB_ is increased, the dispersions turn white due to aggregation of the nanoparticles. Interestingly, by increasing *φ*_1prop_, aggregation occurs at higher values for *c*_CTAB_.

Dynamic light scattering (DLS) measurements confirm the observed trend of particle aggregation (Fig. S6, ESI†). Fig. 3B(ii) shows the DLS intensity averaged size of aqueous NP dispersions with variable *φ*_1prop_ and *c*_CTAB_. The average sizes increase from several tens to hundreds of nanometers by raising *c*_CTAB_. This size increase begins at higher *c*_CTAB_ values as *φ*_1prop_ increases, indicating that higher *φ*_1prop_ enhance the colloidal stability of the nanoparticles.

The colloidal stability dependence on *c*_CTAB_ and *φ*_1prop_ can be explained by electrophoretic mobility (zeta (*ζ*)-potential) measurements. Fig. 3B(ii) shows that the *ζ*-potentials of all dispersions are between −30 and −40 mV at *c*_CTAB_ = 0.1 mM. The negative *ζ*-potential originates from negatively charged silanol and aluminate groups on the Ludox TMA particles.^36,37^ By increasing *c*_CTAB_, the *ζ*-potential becomes less negative, suggesting that positively charged CTAB molecules adsorb on the nanoparticles. However, with increasing *φ*_1prop_, the slopes of the curves begin to increase at higher *c*_CTAB_. Thus, it appears that increasing *φ*_1prop_ decreases the adsorbed amount of CTAB on the nanoparticles for a set value of *c*_CTAB_. For nanoparticles with less CTAB adsorbed, electrostatic repulsion can prevent aggregation.

**Fig. 4:**
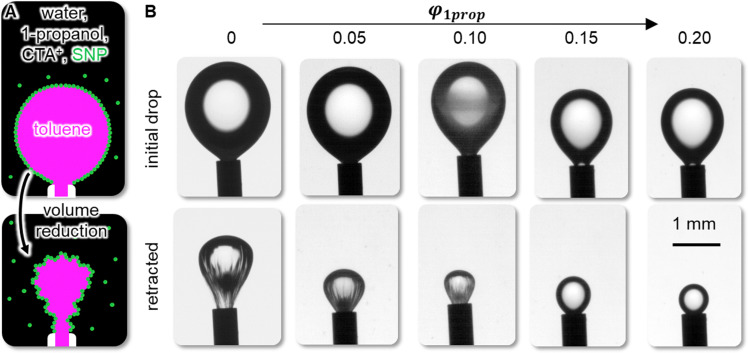
Effect of *φ*_1prop_ on the interfacial rigidity. (A) Schematic depiction of pendant drop retraction experiment. (B) Photographs of pendant droplets before and after volume reduction.

Besides the effect of *φ*_1prop_ on the colloidal stability, *φ*_1prop_ also influences solidification of the oil/water interface, essential for the mechanical stability of the bijel. To probe the effect of *φ*_1prop_ on the mechanics of the oil/water interface, we generate pendant toluene droplets in aqueous dispersions. The aqueous dispersions at pH 3 contain 5 wt% colloidal NPs, 0.2 mM CTAB and variable *φ*_1prop_. The CTAB functionalized NPs self-assemble on the toluene/water interface as schematically depicted in Fig. 4A. Upon volume reduction of the pendant toluene drop *via* a syringe pump, the droplet undergoes deformation as illustrated in Fig. 4A.

Fig. 4B shows photographs before and after volume reduction of the pendant toluene droplets for variable *φ*_1prop_. For 0 ≤ *φ*_1prop_ ≤ 0.10, volume reduction results in buckling and wrinkle formation around the droplet neck. In contrast, for *φ*_1prop_ ≥ 0.15, the pendant droplets do not display significant shape deformations. The buckling and wrinkling at 0 ≤ *φ*_1prop_ ≤ 0.10 qualitatively demonstrates the rigidity of the self-assembled NP layer on the toluene/water interface. Apparently, the interfacial rigidity decreases with increasing *φ*_1prop_, as suggested by the absence of wrinkles for *φ*_1prop_ ≥ 0.15. The decreasing interfacial rigidity with increasing *φ*_1prop_ can result from a reduction of the toluene/water interfacial tension with increasing *φ*_1prop_. A decreasing interfacial tension decreases the interfacial attachment energy of the NPs.^16,38^ Another possible reason for the interfacial rigidity reduction can be decreasing attractive interactions between the interfacial NPs with increasing *φ*_1prop_, as suggested by the aggregation behavior in Fig. 3B. This interpretation is also supported by prior research on the rheology of bijels.^25^ Bijels stabilized by attractive particles have higher storage moduli compared to bijels that are stabilized by interfacial jamming alone.^39^ Nevertheless, to distinguish the different contributions diminishing the interfacial rigidity with increasing *φ*_1prop_, further rheological characterization of the interfacial particle layer is needed.

The shape and boundary structure of the inner water channel in Fig. 2 can now be rationalized by combining insights from miscibility, colloidal stability and interfacial rigidity analysis. The oval shape of the inner channel for *φ*^in^_1prop_ = 0 can be explained by the immiscibility between precursor mixture and inner water channel. Fig. 3A confirms that the precursor mixture phase separates upon addition of small volumes of pure water (*φ*_1prop_ = 0). Fig. 2 shows that for *φ*^in^_1prop_ = 0, STrIPS generates bicontinuous structures growing from the inner water channel into the bijel shell. Both observations suggest that for *φ*^in^_1prop_ = 0, a distinct oil/water interface forms between the inner water channel and the precursor mixture. The interfacial tension of this interface can explain the partially rounded shape of the inner channel for *φ*^in^_1prop_ = 0. Nevertheless, interfacial tension has not been strong enough to shape the water channel fully circular. The oval shape proves that the self-assembly of nanoparticles has rigidified the interface before rounding was completed. Fig. 4 indicates that this interfacial rigidification becomes significant when *φ*_1prop_ drops below 0.1 in the fiber by diffusion. Moreover, Fig. 3B demonstrates that once *φ*_1prop_ decreases sufficiently, also particle aggregation becomes important. Thus, for *φ*^in^_1prop_ = 0, interfacial nanoparticle assembly during phase separation and aggregation has rigidified and structured the boundary between inner water channel and precursor mixture.

However, interfacial self-assembly does not explain the particle crust and angular shape for *φ*^in^_1prop_ = 0.1 and 0.2 in Fig. 2. Here, Fig. 3A shows that precursor mixture and inner water channel are initially miscible. Thus, no continuous liquid–liquid interface is formed between inner water channel and precursor mixture on which nanoparticles could self-assemble. Instead, the particles aggregate in bulk, as suggested by Fig. 3B, resulting in the crust observed in Fig. 2. The angular shape of this rigid crust can result from the square flow cross-section of the injection capillary (Fig. 1C(i)).

Preventing crust formation is essential for unobstructed pore connections between the bijel and the bulk water phase. Fig. 2 shows that unobstructed pore connections can be achieved by raising *φ*^in^_1prop_ ≥ 0.3. Precursor mixtures with *φ*^in^_1prop_ ≥ 0.3 are fully miscible with the inner water channel (Fig. 3A) and the nanoparticles have improved colloidal stability (Fig. 3B). Thus, neither an interface, nor a crust is formed. The partially rounded shape of the inner water channel can result from radial inwards growth of the phase-separated structures. Thus, we can conclude this part with the first important criterium to obtain open bijel pore connections to a bulk water phase: controlling colloidal stability is essential to prevent particle aggregation and crust formation during bijel formation.

**Fig. 5:**
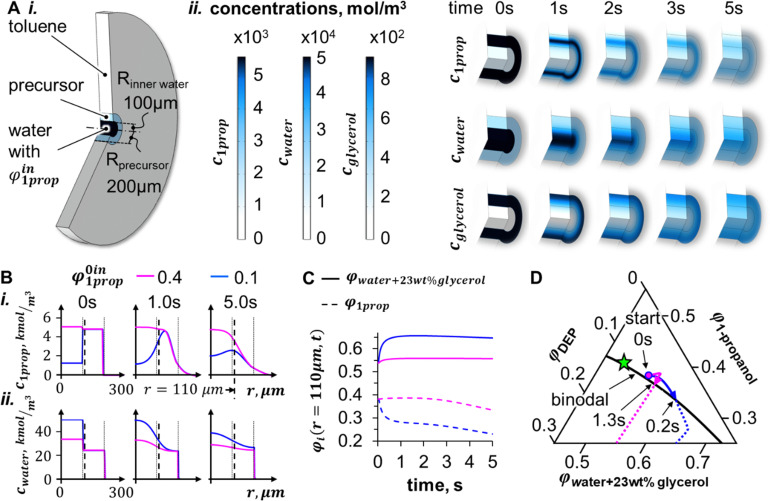
Diffusion model of bijel shell formation. (A)-(i) 3D-cutaway drawing of simulation geometry, (A)-(ii) simulated 3D-surface concentration plots of 1-propanol (*c*_1prop_), water (*c*_water_), and glycerol (*c*_glycerol_) for *φ*^in^_1prop_ = 0.1 over time. (B) Comparison of radial profiles of *c*_water_(*r*) and *c*_1prop_(*r*) for *φ*^in^_1prop_ = 0.1 and 0.4. The thin-dashed vertical lines represent the inner and outer surfaces of the precursor shell. The thick-dashed vertical lines indicate the position *r* = 110 μm. (C) Volume fractions *φ*_i_ at the radial position *r* = 110 μm over time. (D) Simulated composition time evolution at *r* = 110 μm in phase diagram for *φ*^in^_1prop_ = 0.1 (blue) and 0.4 (pink).

After investigating colloidal stability and the shape of the inner water channel, we next discuss the mechanisms of droplet formation for *φ*^in^_1prop_ = 0.1 and 0.2, and the formation of different bicontinuous structures for *φ*^in^_1prop_ = 0 and *φ*^in^_1prop_ ≥ 0.3 in Fig. 2. Droplet formation interrupts the bicontinuous fluid network of the bijel, compromising the connections between the bijel and the adjacent bulk phase. To understand the origins of the droplets, we employ time-dependent diffusion simulations and predict the compositional trajectory the precursor mixture takes in the phase diagram (Fig. S7, ESI†). The model geometry is depicted in Fig. 5A(i) and consists of an outer toluene reservoir, a shell of precursor mixture, and an inner water cylinder with variable *φ*^in^_1prop_.

Fig. 5A(ii) shows 3D surface plots of simulated concentration distributions for *φ*^in^_1prop_ = 0.1. The partial miscibility of the precursor mixture with the inner water stream allows for diffusive exchange of 1-propanol, water, and glycerol. In contrast, towards the outer toluene, only 1-propanol diffusion is significant, since both water and glycerol are sparsely soluble in toluene. The concentration plots in Fig. 5A(ii) show that the 1-propanol concentration (*c*_1prop_) decreases both towards the inner water and the toluene reservoir. Since the water concentration (*c*_water_) is highest in the center, it decreases in the inner water cylinder and increases in the precursor shell. The glycerol concentration (*c*_glycerol_) increases in the inner water and decreases in the precursor shell. In the following, we analyze the concentration distributions in dependence of *φ*^in^_1prop_.

In Fig. 2, droplets appear for *φ*^in^_1prop_ = 0.1 and droplets are absent for *φ*^in^_1prop_ = 0.4. To understand this difference, we compare the concentration evolutions for the two different *φ*^in^_1prop_. The droplets in Fig. 2 are observed at the boundary between precursor mixture shell and inner water cylinder. To represent this boundary, we discuss the concentrations at the radial position *r* = 110 μm. Fig. 5B shows time evolutions of the radial *c*_1prop_(*r*) and *c*_water_(*r*) profiles.

In Fig. 5B(i), *c*_1prop_(110 μm) drops rapidly for *φ*^in^_1prop_ = 0.1 (blue curve), while *c*_1prop_(110 μm) decreases only slightly during 5 seconds for *φ*^in^_1prop_ = 0.4 (pink curve). Also *c*_water_(110 μm) changes (increases) more drastically for *φ*^in^_1prop_ = 0.1 than for *φ*^in^_1prop_ = 0.4, as shown in Fig. 5B(ii). We plot the local volume fractions *φ*_1prop_(110 μm) and *φ*_water+23wt% glycerol_(110 μm) in Fig. 5C (calculated from *c*_water_(*r*), *c*_glycerol_(*r*), *c*_1prop_(*r*)). The curves show that *φ*_1prop_(110 μm) decreases and *φ*_water+23wt% glycerol_(110 μm) increases within less than 1 second for *φ*^in^_1prop_ = 0.1. In contrast, for *φ*^in^_1prop_ = 0.4, *φ*_1prop_(110 μm) only begins to decrease after 3 seconds, while *φ*_water+23wt% glycerol_(110 μm) remains largely unchanged. The different dynamics of *φ*_1prop_(110 μm) and *φ*_water+23wt% glycerol_(110 μm) in dependence of *φ*^in^_1prop_ change the trajectory the composition takes in the phase diagram, as discussed next.

Plotting the results in the phase diagram suggests that the different mass transfer dynamics trigger distinct mechanisms of phase separation at *r* = 110 μm. To plot the simulation results in the phase diagram, we have estimated *φ*_DEP_ = 1 − *φ*_water+23wt% glycerol_ − *φ*_1prop_. The simulations for all *φ*^in^_1prop_ are shown in Fig. S8 (ESI†), but for brevity we focus here only on *φ*^in^_1prop_ = 0.1 and 0.4. The blue curve in the phase diagram of Fig. 5D (*φ*^in^_1prop_ = 0.1) shows that the composition at *r* = 110 μm shifts strongly to the lower right before hitting the binodal curve after 0.2 seconds. The shift occurs because both *φ*_1prop_(110 μm) and *φ*_water+23wt% glycerol_(110 μm) are changing (Fig. 5C). The shift moves the entry point for phase separation away from the critical point (green star). As a result, nucleation and growth of DEP rich droplets in water can occur, similar to what happens when pouring the Greek spirit Ouzo in a glass of water (see schematics in phase diagram of Fig. 1A).^40^

In contrast, the composition of *φ*^in^_1prop_ = 0.4 (pink curve in Fig. 5D) remains in the miscible region for 1.3 seconds before hitting the binodal curve closer to the critical point. The different trajectory originates because only *φ*_1prop_(110 μm) decreases after 3 seconds due to diffusion towards the outer toluene (Fig. 5B(i) and C), while *φ*_water+23wt% glycerol_(110 μm) does not change in the same time span. The different trajectory for *φ*^in^_1prop_ = 0.4 explains the absence of droplets near the inner water/precursor mixture surface in Fig. 2.

It is important to note that the diffusion simulation also predicts nucleation for *φ*^in^_1prop_ = 0 (see Fig. S8, ESI†), but Fig. 2 shows bicontinuous structures for *φ*^in^_1prop_ = 0 near the inner water channel. We can only speculate about the reasons for this discrepancy. The first possible reason is that our model does not predict the correct compositional evolution near the inner water channel/precursor mixture boundary for *φ*^in^_1prop_ = 0. It is plausible that the bicontinuous structures near the inner water channel/precursor mixture restrict the diffusion of water, since they consist of a network of particle stabilized oil channels. This oil barrier can be impermeable to water, but permeable to 1-propanol due to mutual solubility. If only 1-propanol diffuses, the diffusion model predicts that the precursor mixture enters the immiscible region of the phase diagram near the critical point, resulting in spinodal phase separation.

The second possible reason is that rapid quenching for *φ*^in^_1prop_ = 0 can bypass droplet nucleation in the phase diagram. Faster quenching rates occur upon decreasing *φ*^in^_1prop_ due to higher concentration gradients. The simulation predicts that it takes 1.1 seconds for *φ*^in^_1prop_ = 0.2 until the mixture crosses the binodal curve in Fig. 4D. In contrast, for *φ*^in^_1prop_ = 0, it takes only 0.1 seconds until the binodal curve is crossed. Such fast quenching can potentially bypass nucleation into the spinodal region of the phase diagram, giving rise to the bicontinuous structures at the inner water channel for *φ*^in^_1prop_ = 0 in Fig. 2.

With these insights, we conclude this section with the second important criterium to obtain open bijel pore connections towards a bulk water phase: controlling the mass transfer dynamics during STrIPS bijel formation is essential to prevent phase separation *via* nucleation.

### 2.3 Confocal microscopy analysis

**Fig. 6:**
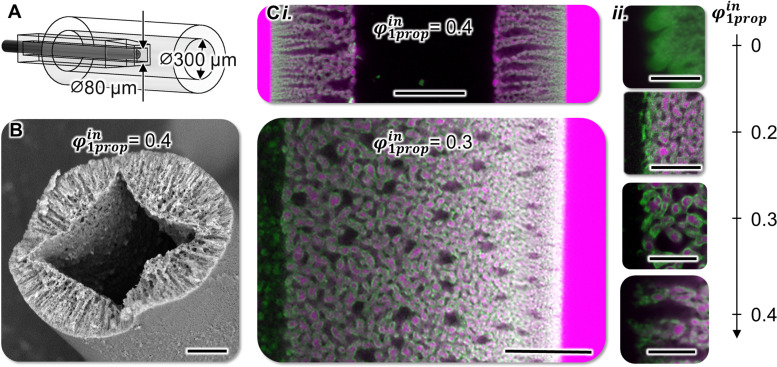
Small-diameter fibers. (A) Computer drawing of microfluidic device used to make small diameter fibers. (B) SEM image of fiber cross-section. Scale bar 20 μm. (C)-(i) CLSM images of equatorial planes of fibers with variable *φ*^in^_1prop_, scale bars 20 μm. (C)-(ii) Magnified CLSM images of boundary region of bijel shell and inner water for variable *φ*^in^_1prop_, scale bars 10 μm.

After discussing the different structures observed in Fig. 2, we employ confocal laser scanning microscopy (CLSM) to visualize the spatial oil, water and particle distribution in the bijel. However, imaging the bijel *via* CLSM is limited by light scattering, restricting how deep below the fiber surface the fluorescence signal can be detected. Due to the large diameter of the fibers in Fig. 2, we are not able to image the equatorial fiber plane *via* CLSM. Therefore, we fabricate smaller diameter fibers with the microfluidic device depicted in Fig. 6A. The inner water stream of variable *φ*^in^_1prop_ flows out of a round cross-section capillary, and the precursor mixture enters the toluene stream through a tapered square cross-section capillary (Fig. S9, ESI†). Fig. 6B shows a SEM image of a smaller diameter fiber with *φ*^in^_1prop_ = 0.4

Surprisingly, in Fig. 6B the inner water channel in the fiber has an angular cross-section, although the capillary used to extrude the water stream has a round cross-section. In contrast, the outer perimeter of the fiber has a round cross-section, despite having flown out of a square capillary. We explain the outer round cross-section of the fiber with the interfacial tension between the precursor mixture and the surrounding toluene. As reasoned before for Fig. 2, the angular shape in the center is possible due to the miscibility of precursor mixture and inner water stream. However, we are not able to explain how the angular shape evolved hydrodynamically and more research is needed to understand this effect in detail.

CLSM images of the equatorial fiber plane in Fig. 6C(i) show that the oil (pink), water (black) and particle (green) networks extend throughout the bijel shell of the fiber. After fiber collection on the rotating turntable in Fig. 1C(iii), we exchange the toluene with a solution of Nile red in hexane to fluorescently label the bijel. Hexane is employed to reduce light scattering during CLSM imaging due to better refractive index match with the water/glycerol solution in the bijel.^35^ For *φ*^in^_1prop_ = 0.4 in Fig. 6C(i), the shell shows radially aligned macrovoids. In contrast, for *φ*^in^_1prop_ = 0.3 the bijel shell is more isotropic with a radial pore size gradient and interspersed water pockets. Understanding the mechanisms generating these different architectures in more detail remains subject of our ongoing research. Intriguingly, the structures in Fig. 6C(i) may facilitate interesting application potentials for bijels to increase the surface area of membranes, batteries, and catalytic reactors.

Fig. 6C(ii) shows magnified insets of the boundary region between inner water and bijel shell in dependence of *φ*^in^_1prop_. For *φ*^in^_1prop_ = 0, the dense green color indicates strongly aggregated silica particles, similar to what we already observed in Fig. 2 for larger diameter fibers. The extent of aggregation decreases for *φ*^in^_1prop_ = 0.2 and aggregation appears to vanish for *φ*^in^_1prop_ ≥ 0.3. In contrast to Fig. 2, the smaller diameter fibers in Fig. 6C(ii) do not show droplets for *φ*^in^_1prop_ = 0.2. A possible explanation is that the shorter diffusion distance has enabled a “by-passing” of droplet nucleation *via* rapid quenching through the binodal region of the phase diagram. Despite this difference, the CLSM images confirm that increasing *φ*^in^_1prop_ to 0.3 opens the pore connections between the bijel and a bulk water phase.

### 2.4 Analysis of the bijel–toluene boundary

**Fig. 7:**
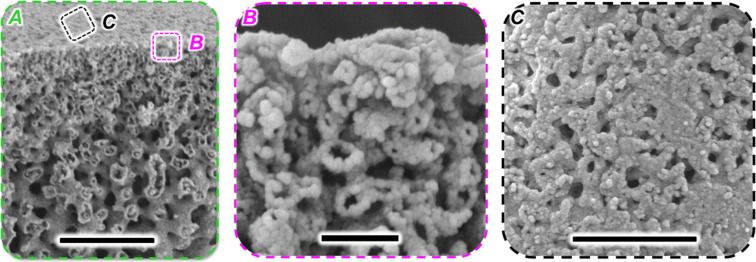
SEM of bijel–toluene boundary. (A) Cross-section of bijel, scale bar 10 μm, (B) magnified side-view of bijel–toluene boundary, scale 1 μm (C) magnification of outer bijel surface pores, scale 5 μm.

In the last section, we analyze the pore connections between the bijel and toluene. Fig. 7 shows magnified SEM images of the bijel–toluene boundary of a TEOS reinforced and dried hollow fiber.

Fig. 7A reveals that the former fluid networks of the bijel decrease in size towards the bijel/toluene boundary at the top. The magnified SEM image of the toluene/bijel boundary in Fig. 7B shows a network of hollow channels below the outer surface of the fiber. Due to their tunnel-like structure, we can identify these channels to correspond to the former oil network of the bijel. But, because of the dense arrangement of particles at the fiber/toluene boundary above, we cannot discern whether the oil tunnels extends to the toluene phase. But, the top view of the bijel/toluene boundary clearly reveals open surface pores in Fig. 7C. Thus, the SEM images strongly suggest that there are open connections between the oil network of the bijel and the toluene phase.

Indirect evidence suggests that the open surface pores on the outer surface of the fiber are indeed connected to the oil network. It is unlikely that the surface pores are connected to the water network, since then an interfacial layer of nanoparticles would self-assemble to close off these pores. Moreover, open connections between the oil network of the bijel and the surrounding toluene are plausible due to diffusion into the bijel from the toluene side. Three different hydrophobic molecules have permeated the bijel from the surrounding oil bath: (i) hexane has reduced the refractive index mismatch in the bijel for Fig. 6C, (ii) Nile red has fluorescently labelled particles and oil in the bijel (also Fig. 6C), and (iii). TEOS has fully cross-linked the bijel (Fig. 2, 6B and 7). The low water solubility of these molecules suggests that they have diffused through the oil network of the bijel. This is confirmed when generating a fiber *via* STrIPS that does not form an internal channel network, but instead separated droplets (Fig. S10, ESI†). The fluorescent dye Nile red was not able to penetrate into the fiber in Fig. S10 (ESI†). Similar to prior work on bijels, the penetration of the bijel by oil soluble species suggests open oil pores on the surface of the bijel.^19,25,26,38,41–43^

## 3. Conclusions

While past research has connected bijels to a single bulk phase,^25,26^ this manuscript introduces dual connections for bijels to two bulk phases. Channel connection control can be obtained during bijel synthesis *via* solvent transfer induced phase separation (STrIPS) through the miscibility of co-flowing bulk phases: polar bulk phases can form connections to the bijel's water domains, whereas apolar bulk phases can form connections to the oil domains. We identify two important criteria for unobstructed connections between the bijel and a polar bulk phase: (i) mitigation of excessive colloidal aggregation to prevent nanoparticle deposit formation, (ii) regulation of diffusive mass transfer to avoid nucleation and growth. Here, both criteria are controlled *via* the addition of 1-propanol to the aqueous bulk phase, enabling open connections between bijel and aqueous bulk phase. At the boundary to an apolar bulk phase, sub-micrometer sized pores facilitate diffusive transport of hydrophobic molecules into the oil network of the bijel. This work investigates dual pore connections for hollow bijel fibers and the results will also be important for bijel sheets.^15,44–46^ Bijels with dual pore connections not only interweave two adjacent bulk phases to increase their surface area, but additionally provide separate access to each of the two microscopic channel networks from opposite sides. In future research, bijel fibers and sheets with dual pore access can potentially be used to enhance mass- and heat-transfer between different materials, *e.g.* between anodes and cathodes in lithium-ion-batteries, or between the feed and permeate sides of filtration membranes.

## 4. Materials and methods

### 4.1 Materials

Hexadecyl-trimethylammonium bromide (CTAB, >99%), 1-propanol (>99.5%), Nile red (for microscopy), tetraethyl orthosilicate (TEOS, >99%), sodium chloride (NaCl, ≥99%) and mineral oil were purchased from Sigma-Aldrich. Silica nanoparticles (Ludox® TMA, spherical, particle diameter of 22 nm) were purchased from Grace. Diethyl phthalate (DEP, 99%), *n*-hexane (99% HPLC), glycerol (>99%), hydrochloric acid (HCl, 37% pure) were received from Acros organics. Toluene (technical grade) was purchased from VWR Chemicals. Octadecyl trichlorosilane (OTS, 94.3%) was purchased from Santa Cruz Biotechnology, Inc.

### 4.2 Preparation of bijel precursor

100 mL of Ludox® TMA dispersion (batch number 20211850133) is brought to pH 3.0 through the addition of 1 M HCl. It is concentrated from 34 wt% to 40 wt% in a rotary evaporator (Heidolph Instruments) at 60 °C and 140 mbar and centrifuged at 3500 rpm for 10 minutes (Allegra X-12R, Beckman Coulter) to remove any particle aggregates. The dispersion is transferred to a dialysis bag (Spectra/Por MWCO 14 000) and placed in a beaker containing 1900 mL MilliQ water at pH 3.0 containing 50 mM NaCl overnight.

To prepare 5 mL of bijel precursor mixture with liquid composition *φ*_DEP_ = 0.079, *φ*_H_2_O_ = 0.432, *φ*_1-propanol_ = 0.383 and *φ*_glycerol_ = 0.106, a nanoparticle loading of 23 wt% Ludox® TMA and a CTAB concentration of 29.2 mM, one mixes the following liquids: 0.350 mL of DEP, 0.645 mL of 200 mM CTAB in 1-propanol, 1.188 mL of 50 wt% glycerol in 1-propanol, 0.317 mL of 1-propanol and 2.500 mL of the Ludox® TMA dispersion prepared as discussed above.

### 4.3 Hollow bijel fiber extrusion

Three syringe pumps (AL-300, world precision instruments) are loaded with syringes containing (1) water-saturated toluene, (2) bijel precursor mixture and (3) the inner fluid. The syringes are connected to the inlets of a microfluidic fiber extrusion device with PTFE tubing (Cole-Parmer). The extrusion device is positioned and secured above a large container (height = 15 cm) filled with water-saturated toluene that is rotated at constant speed using a turntable. The extrusion device is lowered such that the extrusion nozzle is just below the toluene–air interface. The water-saturated toluene, bijel precursor mixture and inner stream are simultaneously flown through the microfluidic device at flowrates of 1 mL min^−1^ for the water-saturated toluene, 10 mL h^−1^ for the bijel precursor mixture and 5 mL h^−1^ for the inner stream (for ∅ 250 μm fibers) or 4 mL h^−1^ for the water-saturated toluene, 2 mL h^−1^ for the bijel precursor mixture and 1 mL h^−1^ for the inner stream (for ∅ 100 μm fibers). The fibers are collected at the bottom of the container.

### 4.4 Scanning electron microscopy analysis

The fibers are transferred into mineral oil containing 3 wt% tetraethyl orthosilicate (TEOS). After 24 hours of reaction at room temperature the fibers are washed with hexane and dried to air. The samples are sputter-coated with a 8 nm thick layer of platinum. The fibers’ cross-section and outer surface are imaged *via* scanning electron microscopy (Phenom ProX, Thermo Fisher Scientific and Gemini 450, Zeiss) using an electron acceleration voltage of 10 kV.

### 4.5 Zeta potential measurements

Aqueous dispersions (2 mL) are prepared by mixing Ludox® TMA nanoparticles (5 wt%, brought to pH 3 through addition of 1 M HCl), 1-propanol (*φ*_1prop_ = 0–0.4) and CTAB (*c*_CTAB_ = 0.1–7 mM). The dispersions that remained colloidally stable overnight are transferred to a disposable folded capillary zeta cell (DTS1070, Malvern Panalytical). The particle size and electrophoretic mobility are measured using a zetasizer (Zetasizer Ultra, Malvern Panalytical). The measurements are done in triplicate and the sample is allowed to equilibrate at 25 °C for 120 seconds before each measurement. The correlation diagrams are recorded and presented in Fig. S6 (ESI†). Fig. S11 (ESI†) presents values for dielectric constant, refractive index and viscosity used for the calculation of zeta potential from the electrophoretic mobility of dispersions with varying *φ*_1prop_.

### 4.6 Pendant drop interfacial rigidity experiments

A pendant drop tensiometer (Dataphysics OCA25) is used to form droplets of 4–6 μL water-saturated toluene in aqueous Ludox TMA dispersions (5 wt%, 0.2 mM CTA+, pH 3). Both the toluene and the water phase contain 0/5/10/15/20 vol% 1-propanol. The toluene droplets are equilibrated for 1 min in the aqueous phase and after this, retracted at 0.3–0.5 μL s^−1^. The shrinkage process is recorded *via* a camera at 25 fps (Thorlabs CS165MU/M camera).

### 4.7 Confocal microscopy analysis

The fibers are transferred to a sample container that is crafted from a glass cylinder (diameter ∼0.5 cm) attached onto a microscopy coverslip (22 × 22 × 0.15 mm; Menzel-Gläser) with epoxy glue (Liqui Moly 6183). The toluene is replaced with water-saturated hexane containing Nile red. The fibers are analyzed by inverted confocal laser scanning microscopy (Stellaris 5, Leica Microsystems). The sample is excited using two separate lasers operating at wavelengths of 488 nm and 561 nm. Fluorescence emission is detected at wavelength ranges spanning from 500–550 nm and 600–700 nm.

## Data availability

The data supporting this article have been included as part of the ESI.†

## Conflicts of interest

The authors declare no competing financial interest.

## Supplementary Material

MH-011-D4MH00495G-s001

## References

[cit1] Tenney S., Remmers J. (1963). Comparative quantitative morphology of the mammalian lung: diffusing area. Nature.

[cit2] Zhang Z., Wang X., Yan Y. (2021). A review of the state-of-the-art in electronic cooling. e-Prime-Advances in Electrical Engineering, Electronics and Energy.

[cit3] Madou M. J. , Fundamentals of microfabrication: the science of miniaturization, CRC Press, 2018

[cit4] Fernández-Rico C. (2024). *et al.*, Elastic microphase separation produces robust bicontinuous materials. Nat. Mater..

[cit5] Huang C. (2017). *et al.*, Bicontinuous structured liquids with sub-micrometre domains using nanoparticle surfactants. Nat. Nanotechnol..

[cit6] Di Vitantonio G. (2018). *et al.*, Robust bijels for reactive separation *via* silica-reinforced nanoparticle layers. ACS Nano.

[cit7] Cha S. (2019). *et al.*, Bicontinuous Interfacially Jammed emulsion Gels (bijels) as Media for enabling enzymatic Reactive separation of a Highly Water Insoluble substrate. Sci. Rep..

[cit8] Witt J., Mumm D., Mohraz A. (2016). Microstructural tunability of co-continuous bijel-derived electrodes to provide high energy and power densities. J. Mater. Chem. A.

[cit9] McDevitt K. M., Mumm D. R., Mohraz A. (2019). Improving cyclability of ZnO electrodes through microstructural design. ACS Appl. Energy Mater..

[cit10] Gross S. J. (2022). *et al.*, Alleviating expansion-induced mechanical degradation in lithium-ion battery silicon anodes *via* morphological design. Extreme Mech. Lett..

[cit11] Cai D. (2018). *et al.*, Direct transformation of bijels into bicontinuous composite electrolytes using a pre-mix containing lithium salt. Mater. Horiz..

[cit12] Witt J. A., Mumm D. R., Mohraz A. (2013). Bijel reinforcement by droplet bridging: a route to bicontinuous materials with large domains. Soft Matter.

[cit13] Thorson T. J. (2019). *et al.*, Bijel-templated implantable biomaterials for enhancing tissue integration and vascularization. Acta Biomater..

[cit14] Thorson T. J., Botvinick E. L., Mohraz A. (2018). Composite bijel-templated hydrogels for cell delivery. ACS Biomater. Sci. Eng..

[cit15] Wang T. (2023). *et al.*, Bioinspired Switchable Passive Daytime Radiative Cooling Coatings. ACS Appl. Mater. Interfaces.

[cit16] Siegel H. (2022). *et al.*, Synthesis and Polyelectrolyte Functionalization of Hollow Fiber Membranes Formed by Solvent Transfer Induced Phase Separation. ACS Appl. Mater. Interfaces.

[cit17] Haase M. F. (2017). *et al.*, Multifunctional nanocomposite hollow fiber membranes by solvent transfer induced phase separation. Nat. Commun..

[cit18] Stratford K. (2005). *et al.*, Colloidal jamming at interfaces: A route to fluid-bicontinuous gels. Science.

[cit19] Herzig E. M. (2007). *et al.*, Bicontinuous emulsions stabilized solely by colloidal particles. Nat. Mater..

[cit20] Karan S., Jiang Z., Livingston A. G. (2015). Sub–10 nm polyamide nanofilms with ultrafast solvent transport for molecular separation. Science.

[cit21] Werner J. (2018). *et al.*, Block copolymer derived 3-D interpenetrating multifunctional gyroidal nanohybrids for electrical energy storage. Energy Environ. Sci..

[cit22] Pera-Titus M. (2015). *et al.*, Pickering interfacial catalysis for biphasic systems: from emulsion design to green reactions. Angew. Chem., Int. Ed..

[cit23] Di Vitantonio G. (2021). *et al.*, Fabrication and application of bicontinuous interfacially jammed emulsions gels. Appl. Phys. Rev..

[cit24] Cates M. E., Clegg P. S. (2008). Bijels: a new class of soft materials. Soft Matter.

[cit25] Lee M. N. (2013). *et al.*, Making a robust interfacial scaffold: Bijel rheology and its
link to processability. Adv. Funct. Mater..

[cit26] Haase M. F., Stebe K. J., Lee D. (2015). Continuous fabrication of hierarchical and asymmetric bijel microparticles, fibers, and membranes by solvent transfer-induced phase separation (STRIPS). Adv. Mater..

[cit27] Ronco C., Clark W. R. (2018). Haemodialysis membranes. Nat. Rev. Nephrol..

[cit28] Liu Y., Zhu Y., Cui Y. (2019). Challenges and opportunities towards fast-charging battery materials. Nat. Energy.

[cit29] Plankensteiner N. (2023). *et al.*, Photovoltaic–Electrolyzer System Operated at> 50 mA cm− 2 by Combining Large-Area Shingled Silicon Photovoltaic Module with High Surface Area Nickel Electrodes for Low-Cost Green H2 Generation. Sol. RRL.

[cit30] Forner-Cuenca A. (2015). *et al.*, Engineered water highways in fuel cells: radiation grafting of gas diffusion layers. Adv. Mater..

[cit31] Haase M. F. , *et al.*, Bijels Formed by Solvent Transfer-induced Phase Separation, in Bijels, 2020, pp. 137–166 10.1039/c9sm00289h 30932124

[cit32] Boakye-Ansah S., Schwenger M. S., Haase M. F. (2019). Designing bijels formed by solvent transfer induced phase separation with functional nanoparticles. Soft Matter.

[cit33] Boakye-Ansah S., Khan M. A., Haase M. F. (2020). Controlling surfactant adsorption on highly charged nanoparticles to stabilize bijels. J. Phys. Chem. C.

[cit34] Khan M. A. (2022). *et al.*, Nanostructured, Fluid-Bicontinuous Gels for Continuous-Flow Liquid–Liquid Extraction. Adv. Mater..

[cit35] Sprockel A. J. (2023). *et al.*, Fabrication of bijels with sub-micron domains *via* a single-channel flow device. Colloids Surf., A.

[cit36] Iler R. K. , The Colloid Chemistry of Silica and Silicates, Cornell University Press, 1955, p. 324

[cit37] Alexander G. B. and Iler R. K., Process for modifying the properties of a silica sol and product thereof, 1959

[cit38] Binks B. P. and Horozov T. S., Colloidal particles at liquid interfaces, Cambridge University Press, 2006

[cit39] Sanz E. (2009). *et al.*, Colloidal gels assembled *via* a temporary interfacial scaffold. Phys. Rev. Lett..

[cit40] Haase M. F. (2016). *et al.*, In situ mechanical testing of nanostructured bijel fibers. ACS Nano.

[cit41] Vitale S. A., Katz J. L. (2003). Liquid droplet dispersions formed by homogeneous liquid− liquid nucleation:“The Ouzo Effect”. Langmuir.

[cit42] Tavacoli J. W. (2011). *et al.*, Novel, Robust, and Versatile Bijels of Nitromethane, Ethanediol, and Colloidal Silica: Capsules, Sub-Ten-Micrometer Domains, and Mechanical Properties. Adv. Funct. Mater..

[cit43] Bai L. (2015). *et al.*, Dynamics and rheology of nonpolar bijels. Soft Matter.

[cit44] Bray A. J. (2002). Theory of phase-ordering kinetics. Adv. Phys..

[cit45] Siegel H. (2024). *et al.*, Roll-to-Roll Fabrication of Bijels *via* Solvent Transfer Induced Phase Separation (R2R-STrIPS). Adv. Mater. Technol..

[cit46] Li J., Sun H., Wang M. (2020). Phase inversion-based technique for fabricating bijels and bijels-derived structures with tunable microstructures. Langmuir.

