# Supplementary material for: Dual access to the fluid networks of colloid-stabilized bicontinuous emulsions through uninterrupted connections
Source: Mater Horiz. 2024 Jul 25;11(20):4987–97. doi: 10.1039/d4mh00495g (PMC11472867; doi:10.1039/d4mh00495g)
Supplement: MH-011-D4MH00495G-s001 [file MH-011-D4MH00495G-s001.pdf]

Supplementary Information (SI) for Materials Horizons.
This journal is © The Royal Society of Chemistry 2024

Supporting Information

Maximizing surface area between two liquids with particle-stabilized, bicontinuous emulsions

*Mariska de Ruiter[1], Meyer T. Alting[1], Henrik Siegel[1], Martin F. Haase[1]**

[1] Van't Hoff Laboratory of Physical and Colloid Chemistry, Department of Chemistry, Debye Institute for Nanomaterials Science, Utrecht University, Utrecht, The Netherlands

* Correspondence: m.f.haase@uu.nl

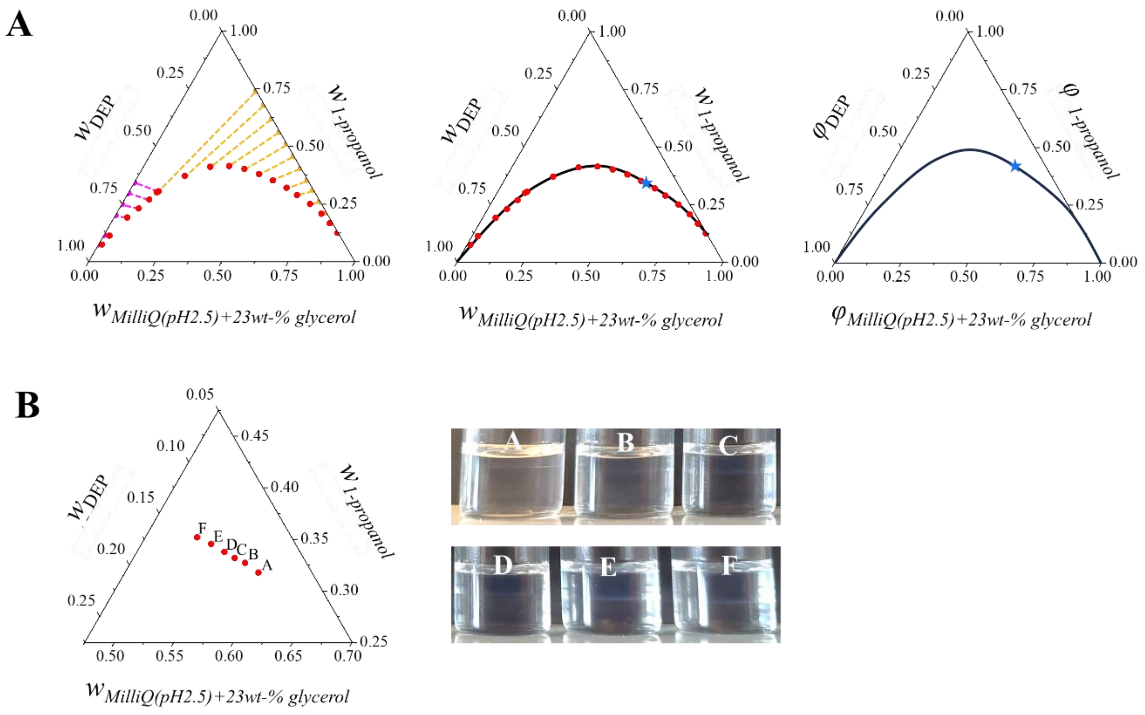

Figure S1. Determination of (A) binodal curve and (B) critical point.

Figure S1A shows the measurement of the binodal curve in the ternary phase diagram of diethyl phthalate (DEP), 1-propanol and water at pH 2.5 containing 23 wt-% glycerol (here denoted as aqueous phase) by turbidimetry. In brief, DEP is added to a miscible mixture of 1-propanol with aqueous phase (orange dashed lines) or aqueous phase is added to a miscible mixture of 1-propanol with DEP (pink dashed lines) until the mixture becomes turbid. All phases are measured in weight fractions (w_i). Weight fractions are converted into volume fractions (φ_i) using the respective densities.

The critical point is determined using a method based on the Lever rule and tie lines along the binodal curve. In summary, an immiscible composition will phase separate in a water-rich and oil-rich phase according to the tie lines with respective volumes V_1 and V_2 [1]. The volumetric ratio between the two phases relates to the volumetric compositions at the ends of the tie line. Near the critical point, the volumes of both phases of the phasewill be the same. **Figure S1B** shows photographs of six different immiscible compositions that have phase separated. It can be seen that the volumes of both phases are the same in vial E. Here we found the critical point to be equal to composition E with w_{DEP} = 0.121, $w_{\text{aqueous phase}}$ = 0.544 and $w_{\text{1-propanol}}$ = 0.345.

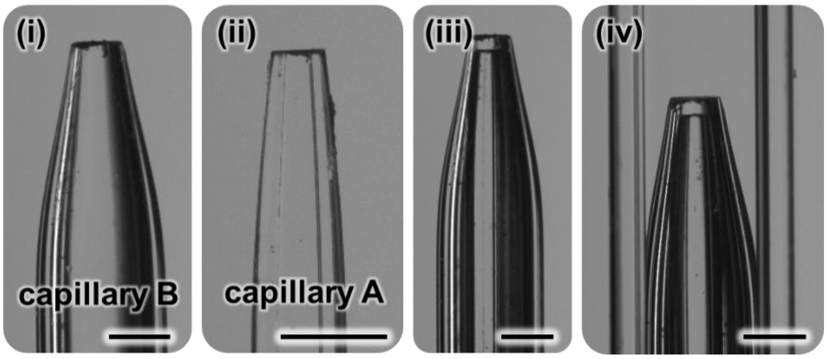

Figure S2. Assembly of a microfluidic device for fabrication of a ø250-300 µm hollow fiber. Scale bars are 500 µm.

Glass capillaries are purchased from Vitrocom. Three different capillaries are used with the following specifications: capillary A – square, 0.2 mm ID / 0.4 mm OD; capillary B – round, 0.58 mm ID / 1.00 mm OD; capillary C – square, 1.05 mm ID / 1.4 mm OD.

(i) Capillary B is tapered using a micropipette puller (Model P-1000, Sutter Instrument) and cut with a scoring tile (Sutter) to give an orifice of ~ 300 µm. (ii) Capillary A is tapered using a micropipette puller and cut with a scoring tile (Sutter) to give an orifice of ~ 170 µm. (iii) Capillary A is inserted in capillary B until the corners of capillary A jam in the tapered walls of capillary B. The two nozzles should be separated by a distance of ~ 100 µm. (iv) Capillary C is glued to a glass microscopy slide using epoxy glue (Liqui Moly 6183). A lighter is used to heat the glass such that capillary A can be curved upwards. The capillary combination of A and B is inserted in capillary C and glued to the microscopy slide with epoxy glue. Using the same epoxy glue, three dispensing needles are glued above the nozzles of capillaries A, B and C as inlets. The assembly is embedded in epoxy glue to prevent leakage. The glue is left to harden for >4 hours before the glass capillaries are treated with 3 wt-% of OTS in mineral oil for 16 hours at room temperature.

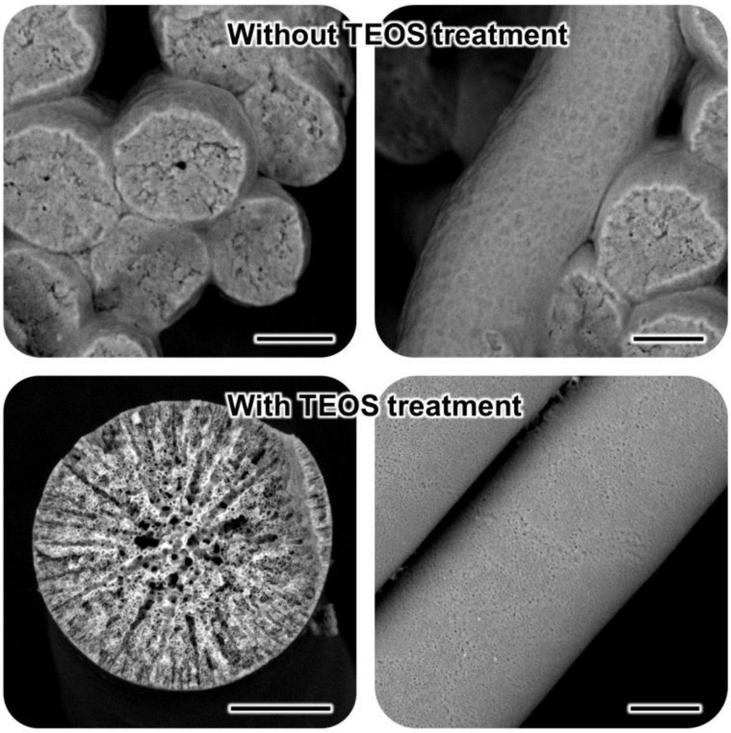

Figure S3. Scanning electron micrographs of dried bijel fibers. For the fibers shown in the top row, no TEOS treatment was applied. The porous bijel structure collapses upon drying. For the fibers shown in the bottom row, the applied TEOS treatment preserves the porous bijel structure upon drying. Scale bars are 20 µm.

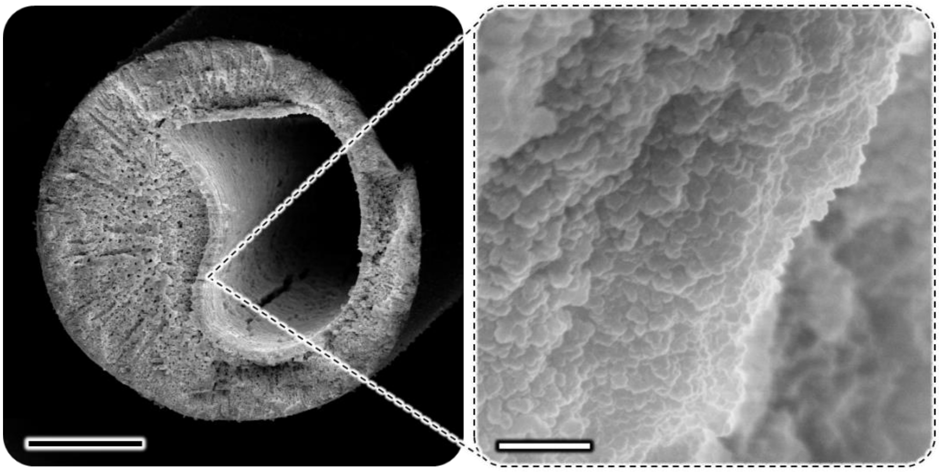

Figure S4. High resolution scanning microscopy of a hollow bijel fiber that was extruded with an inner stream of water with φ_{1prop}^{in} = 0.1. The inset shows that the crust is composed of densely packed nanoparticles. It appears that the nanoparticles are covered by a layer of additional silica as a result of the TEOS treatment. Black scale bar is 100 µm, white scale bar is 500 nm.

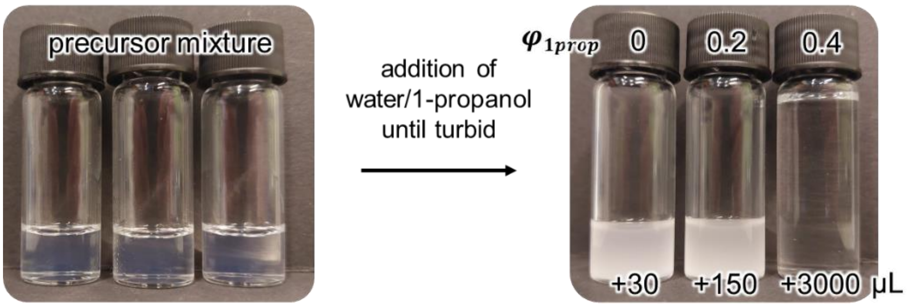

Figure S5. Investigation of the miscibility of a bijel precursor mixture with water/1-propanol mixtures. This experiment was performed with 3 vials each containing 1 mL of a DEP/1-propanol/water mixture. The mixtures have a composition identical to that of a bijel precursor mixture, but for simplicity glycerol is replaced by water and no nanoparticles or surfactant are added. To every vial, a water (pH 3)/1-propanol mixture of varying 1-propanol volume fraction φ_{1prop} was added until the mixture became turbid. The volume that was required for the precursor mixture to become turbid is found to increase with increasing φ_{1prop}. For φ_{1prop}= 0.4 the precursor mixture never becomes turbid.

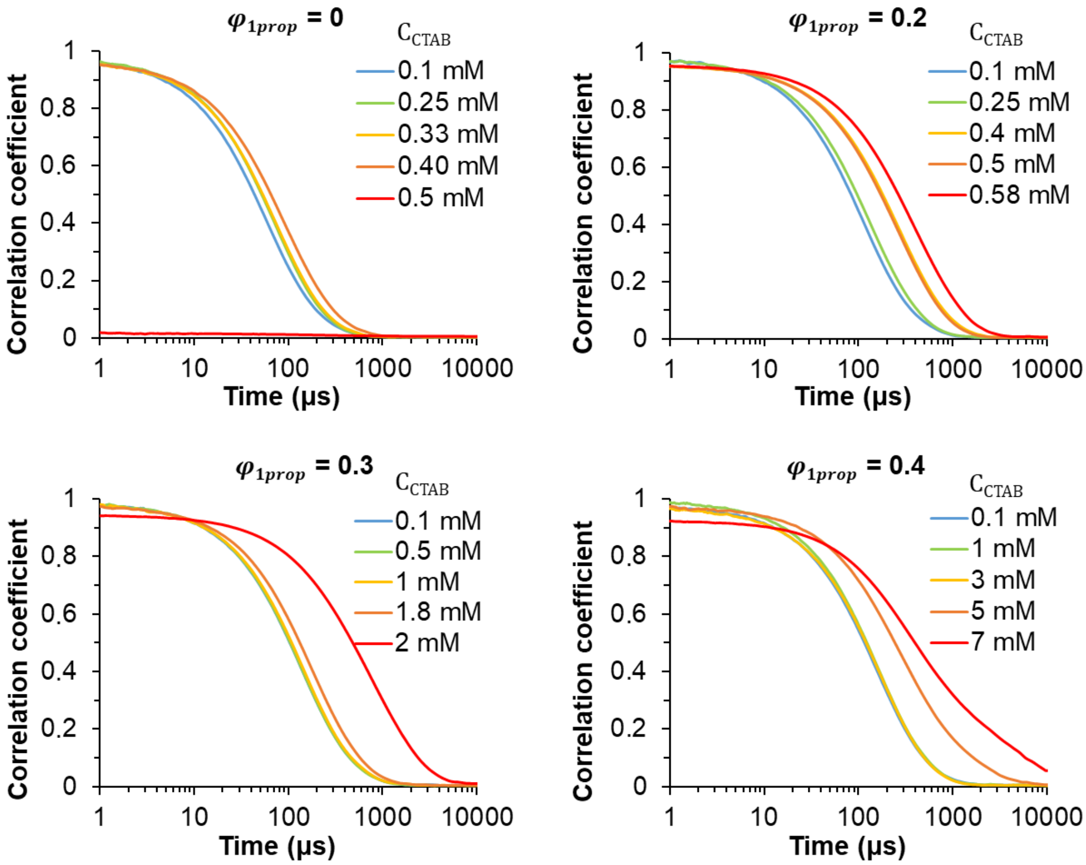

Figure S6. Correlation curves for dynamic light scattering (DLS) measurements of aqueous dispersions of the Ludox TMA NPs.

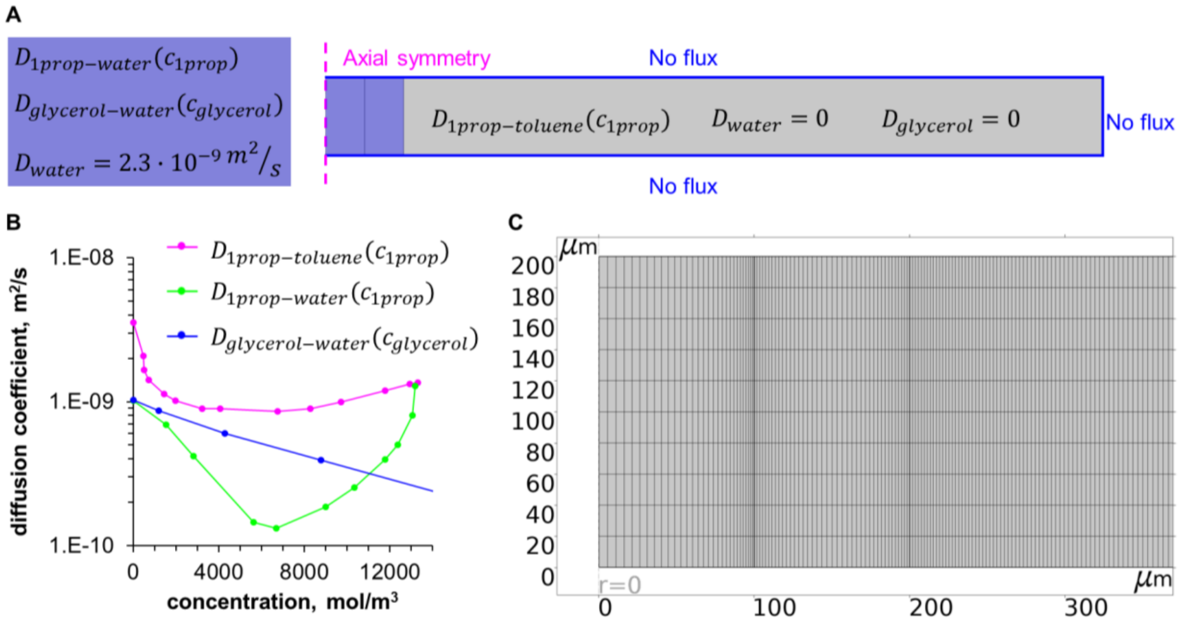

Figure S7. Diffusion simulation. The simulation is configured in COMSOL 5.6 via 2D-axisymmetric geometry. We use a time dependent physics model "Transport of Diluted Species" with 3 concentration variables (c_{1prop}, c_{water}, $c_{glycerol}$). Initial values for these concentrations are fixed in the three different model domains (inner water, precursor, outer toluene). The outer toluene radius is 2000 µm. **A** Model geometry with boundary conditions and definition of diffusion coefficients in the different regions of the simulation (inner water and precursor shell in blue, toluene in gray). **B** Concentration dependent diffusion coefficients employed for the simulation (the water diffusion coefficient was taken as a constant value). In COMSOL, the diffusion coefficients are defined under global definitions as interpolation functions. In the Transport of Diluted Species physics settings, three different transport property tabs are created, in which the interpolation functions are employed in dependence of the concentrations c_{1prop}, c_{water}, $c_{glycerol}$. **C** Simulation mesh used for the numerical solution of the time dependent diffusion model. The time dependent solver uses a relative tolerance of 0.001. The output times are given in seconds by "range(0,0.01,2) range(2.5,0.5,10) range(11,1,20) range(30,10,100)". To measure the concentration at the radial position 110 µm, a domain point probe is created under definitions, with three point probe expressions (c_{1prop}, c_{water}, $c_{glycerol}$).

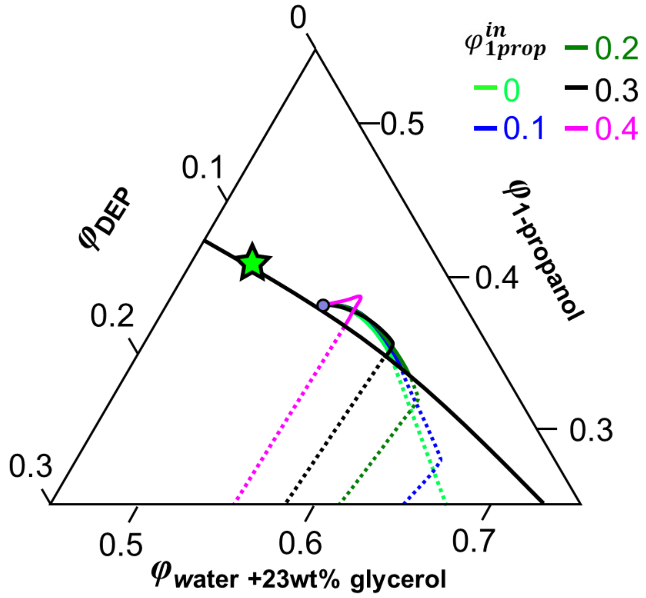

Figure S8. Simulated composition time evolution at r =110 µm in phase diagram for φ^{in}_{1prop} = 0 – 0.4. The black curve represents the binodal line and the green star represents the critical point.

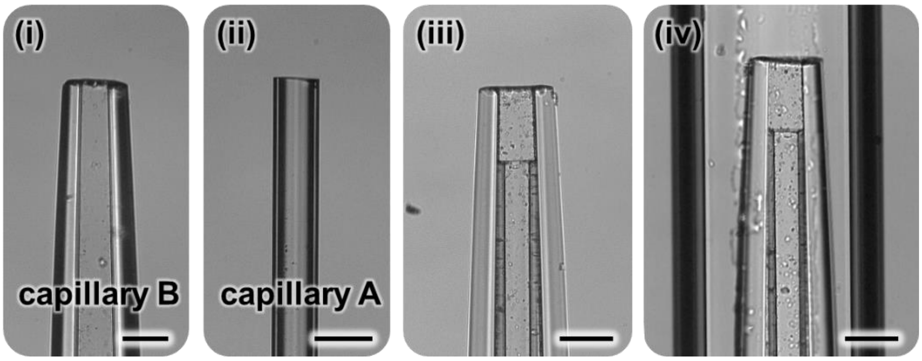

Figure S9. Assembly of a microfluidic device for fabrication of a ø100 µm hollow fiber. Scale bars are 100 µm.

Glass capillaries are purchased from Vitrocom. Three different capillaries are used with the following specifications: capillary A – round, 50 µm ID / 80 µm OD; capillary B – square, 100 µm ID / 200 µm OD; capillary C – round, 300 µm ID / 400 µm OD.

(i) Capillary B is tapered using a micropipette puller (Model P-1000, Sutter Instrument) and cut with a scoring tile (Sutter) to give an orifice of ~75 µm. (ii) Capillary A is cut with a scoring tile to give a straight orifice of 80 µm. (iii) Capillary A is inserted in capillary B until the orifice of capillary A jams into the walls of capillary B. The two nozzles should be separated by a distance of ~ 100 µm. Capillary C is glued to a glass microscopy slide using epoxy glue (Liqui Moly 6183). (iv) The capillary combination of A and B is inserted in capillary C and glued to the microscopy slide with epoxy glue.

Dispensing needles are glued above the nozzles of capillaries A, B and C as inlets. The assembly is embedded in epoxy glue to prevent leakage. The glue is left to harden for >4 hours before the glass capillaries are treated with 3 wt-% of OTS in mineral oil for 16 hours at room temperature.

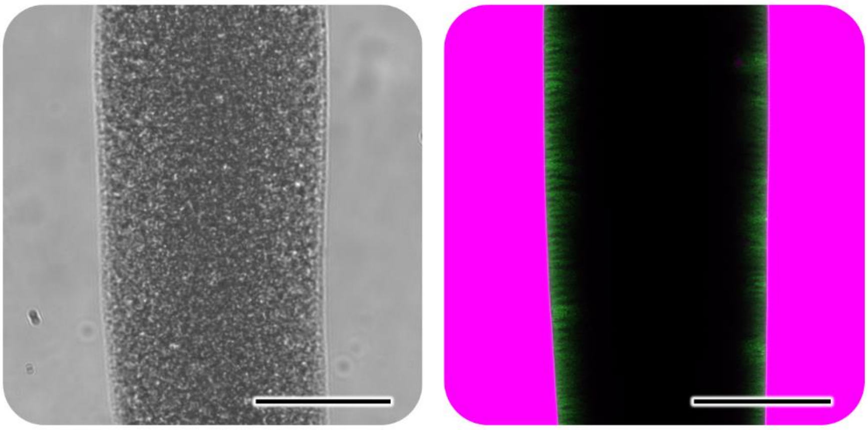

Figure S10. Brightfield (left) and confocal (right) imaging of a fiber composed of unconnected oil droplets in a continuous water shell. The fluorescent dye does not penetrate into the oil droplets. Scale bars are 50 µm.

φ_{1prop}	Dielectric constant	Refractive index	Viscosity (mPa·s)
0	78.2	1.3365	0.895
0.2	66.6	1.3514	1.836
0.3	60.8	1.3584	2.226
0.4	55.0	1.3637	2.497

Figure S11. Values for dielectric constant, refractive index and viscosity used for the calculation of zeta potential from the electrophoretic mobility of aqueous Ludox TMA dispersions with varying φ_{1prop}. The values for dielectric constant are approximated by linear interpolation of the known dielectric constants of water (78.2) and 1-propanol (20.1). The values for refractive index are measured using a refractometer. The values for viscosity are taken from reference [2].

References

1. Khan, M.A., et al., *Nanostructured, Fluid-Bicontinuous Gels for Continuous-Flow Liquid–Liquid Extraction.* Advanced Materials, 2022. **34**(18): p. 2109547.
2. Mikhail, S. and W. Kimel, *Densities and Viscosities of 1-Propanol-Water Mixtures.* Journal of Chemical and Engineering Data, 1963. **8**(3): p. 323-328.
